# An Internet-Based Education Program for Human Papillomavirus Vaccination Among Female College Students in Mainland China: Application of the Information-Motivation-Behavioral Skills Model in a Cluster Randomized Trial

**DOI:** 10.2196/37848

**Published:** 2022-09-30

**Authors:** Mingyu Si, Xiaoyou Su, Yu Jiang, Wenjun Wang, Xi Zhang, Xiaofen Gu, Li Ma, Jing Li, Shaokai Zhang, Zefang Ren, Yuanli Liu, Youlin Qiao

**Affiliations:** 1 School of Population Medicine and Public Health Chinese Academy of Medical Sciences and Peking Union Medical College Beijing China; 2 School of Nursing Jining Medical University Jining China; 3 Key Laboratory of Carcinogenesis and Translational Research (Ministry of Education) Beijing Office for Cancer Prevention and Control Peking University Cancer Hospital & Institute Beijing China; 4 Affiliated Tumor Hospital Xinjiang Medical University Urumqi China; 5 School of Public Health Dalian Medical University Dalian China; 6 West China School of Public Health West China Forth Hospital Sichuan University Chengdu China; 7 Henan Cancer Hospital Affiliated Cancer Hospital of Zhengzhou University Zhengzhou China; 8 School of Public Health Sun Yat-sen University Guangzhou China; 9 School of Health Policy and Management Chinese Academy of Medical Sciences and Peking Union Medical College Beijing China

**Keywords:** human papillomavirus vaccination, internet-based education, information-motivation-behavioral skills model, female college students, China

## Abstract

**Background:**

Patients diagnosed with cervical cancer in the last 2 decades were mainly young females. Human papillomavirus (HPV) vaccination is the most radical way to prevent HPV infection and cervical cancer. However, most female college students in mainland China have not yet been vaccinated, and their relevant knowledge is limited. Theory-based education delivered via the internet is a potentially accessible and useful way to promote HPV vaccination among this population.

**Objective:**

This 3-month follow-up study intended to identify the feasibility and efficacy of an information-motivation-behavioral skills (IMB) model–based online intervention for promoting awareness and willingness regarding HPV vaccination among female college students.

**Methods:**

A 7-day online HPV education program for female college students in mainland China was developed using a cluster randomized trial design. Recruitment and questionnaire surveys were performed online without face-to-face contact. SPSS 23.0 was used for statistical analysis. The chi-square test and *t* test were used to compare differences in qualitative and continuous variables between intervention and control groups. The generalized estimating equation was used to test the effectiveness of the intervention with a consideration of the time factor.

**Results:**

Among 3867 participants, 102 had been vaccinated against HPV before the study (vaccination rate of 2.6%). A total of 3484 participants were followed up after the baseline survey, with no statistical difference in the loss rate between the intervention and control groups during the intervention and follow-up periods. At different follow-up time points, HPV-related knowledge, and the motivation, behavioral skills, and willingness regarding HPV vaccination were higher in the intervention group than in the control group. HPV-related knowledge was statistically different between the 2 groups, while the motivation, behavioral skills, and willingness regarding HPV vaccination only showed statistical differences right after the intervention, reaching a peak right after the intervention and then gradually reducing over time. Furthermore, there was no statistical difference in the HPV vaccination rate between the 2 groups.

**Conclusions:**

IMB model–based online education could be a promising way to increase the HPV vaccination rate and reduce the burden of HPV infection and cervical cancer among high-risk female college students in China.

**Trial Registration:**

Chinese Clinical Trial Registry ChiCTR1900025476; http://www.chictr.org.cn/showprojen.aspx? proj=42672

**International Registered Report Identifier (IRRID):**

RR2-DOI:10.1186/s12889-019-7903-x

## Introduction

### Background

Cervical cancer, a serious infectious disease mostly caused by high-risk human papillomavirus (HPV) types, is the fourth most common reason worldwide for tumor-related hospital admission in women [1,2]. In 2018, in China, cervical cancer resulted in 48,000 deaths and 106,000 cases [1]. Previous studies found that young women were at higher risk of HPV infection, and showed elevated HPV infection problems owing to their age and the sexually transmitted nature of the virus [3-5]. Female college students, most of whom were ready for sexual debut and had relatively low awareness of disease prevention, similarly demonstrated HPV infection problems right after starting college [4,6].

At the end of 2020, China initiated a program for cancer prevention jointly issued by 10 ministries, including the National Health Commission, and joined in the action of the “global strategy to accelerate the elimination of cervical cancer” advocated by the World Health Organization [7-9]. Since the approval of preventive HPV vaccines in mainland China in 2016, the HPV vaccination rate has remained poor, highlighting the importance of understanding the reasons for vaccination refusal and the ways to promote HPV vaccination among young populations [6,10].

Improving the HPV vaccination rate and vaccination willingness is the most salient way to eliminate cervical cancer [11,12]. Most published studies on the willingness to undergo HPV vaccination among Chinese college students were conducted before the availability of HPV vaccines in China [13-15]. Information about the actual rate of HPV vaccination and willingness to undergo HPV vaccination after the approval of HPV vaccines is scarce. Interventions guided by health behavioral theories were widely applied in previous studies [16-19]. A review conducted in 2018 identified 70 scientific articles providing supportive evidence that education guided by health behavioral theories was effective in promoting HPV vaccination [20]. However, only a few interventions on the perceptions of HPV vaccination have been published in China, and they involved limited sample sizes and did not involve health behavioral theories [21-23].

Currently, in China, there is a free HPV vaccination program in pilot regions among school girls less than 14 years old [24], but there are no effective strategies to improve HPV vaccine coverage among female college students who are at higher risk of HPV infection and have been suggested to be a “catch-up” population for HPV vaccination in many other countries. The information-motivation-behavioral skills (IMB) model is one of the commonly used theories on health promotion. To our knowledge, no previous study has been performed to evaluate IMB model–based online education for HPV vaccination among female college students from different regions in mainland China, with a follow-up duration of 3 months.

### Objectives

This study has the following 3 objectives: (1) to evaluate the feasibility and acceptability of the IMB model–based online intervention; (2) to examine whether and how this intervention improves HPV vaccination; and (3) to identify the barriers and facilitators of HPV vaccination among female college students in mainland China.

## Methods

### Study Design

The protocol for this study was published before the interventions were carried out [25]. Briefly, this study was a multicenter 2-arm cluster randomized trial. First-year female college students from 7 universities from different geographical locations in mainland China were recruited and randomly assigned 1:1 to either an IMB model–based intervention group or a waitlist control group based on their class. The intervention group received online education to promote the willingness to undergo HPV vaccination for 7 consecutive days, which was guided by the IMB model and conducted on a communication platform called DingTalk (Alibaba Group). The intervention group was compared with the waitlist control group in terms of the perceptions of HPV vaccination and willingness to undergo HPV vaccination. Recruitment of the study participants began in February 2020, and data collection was completed in July 2020. The whole process of participant recruitment, data collection, and intervention was conducted online, which coincided with the outbreak of COVID-19 in China, when Chinese college students were all self-isolated at home. The CHERRIES (Checklist for Reporting Results of Internet E-Surveys) checklist was used to guide the reporting of our web-based survey [26]. The CONSORT-EHEALTH (Consolidated Standards of Reporting Trials of Electronic and Mobile Health Applications and Online Telehealth) checklist is presented in Multimedia Appendix 1 [27].

### Recruitment and Eligibility

Participating students were recruited through notices on campus or advertisements in social media groups, posted in advance by partner teachers. Interested participants scanned the QR code on the notices or advertisements to fill in their class name and provide informed consent regarding the research objectives, requirements, procedures, benefits, and other study-related information. Potential participants were assessed for eligibility before the baseline survey. The eligibility criteria for the study were as follows: (1) female sex; (2) age ≥18 years; (3) first-year college student; (4) no vaccination contraindications; and (5) accessibility to computers or smartphones. Enrolled participants were given a link to either the intervention group or the control group that had already been set up in advance in DingTalk.

### Randomization and Blinding

In order to facilitate the management of participants and reduce loss of follow-up, enrolled participants were randomly assigned by class to the intervention and control groups. The investigator in each college, who was blinded to the identity of the participants, used computer software (Excel program, Microsoft Corp) to generate a series of random numbers for both the first-year Arts and Science major classes. Eligible participants were identified as either the intervention group or the control group based on the class name they entered when they scanned the QR code on the recruiting information. Participants, data analysts, and investigators were all blinded to the randomized allocation, and only the research assistants in each center were aware of the allocation.

### IMB Model–Based Intervention

#### Theoretical Framework

The theoretical framework used to guide this online intervention was the IMB model [28]. This theory assumes that a person with rich knowledge will have the intention to practice healthy behaviors when he/she has motivation, the ability/skills to complete healthy behaviors, and self-efficacy, and the intention will be easily transformed into actual practice when objective conditions permit [19,29]. Therefore, we developed 2-day materials to popularize HPV knowledge, 2-day materials on situational stories to motivate participants to vaccinate themselves against HPV, and 3-day materials on objective skills with self-decision making, self-efficacy, and objective conditions for making an appointment and receiving the HPV vaccine. The above intervention materials were designed as online readable texts or videos that could be easily accessed by the target population.

#### Intervention Materials

The intervention materials were developed by the research team based on the IMB model, and were uploaded and shared via the DingTalk platform by research assistants at each center. Upon randomization, the intervention group accessed the materials by scanning the QR code on each of the cover pages of the educational materials. During the daily intervention, we included quizzes on each day’s topics to check and consolidate the knowledge gained by the participants. It took about 10-15 minutes for the participants to read and learn the materials, depending on their learning ability. The forms, contents, and corresponding purposes of the IMB model–based education in this intervention are presented in Table 1.

**Table 1:** Cover pages, contents, and purposes of the information-motivation-behavioral skills model–based 7-day education.

Serial number	Cover page	Content	Examples of the quizzes	Purpose
1	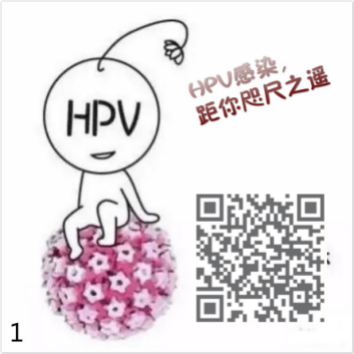	General facts about HPV^a^, including HPV infection and related diseases. For example, there are more than a hundred different types of HPV, some of which are high risk and linked to the development of cancers, including cervical cancers.	1. What is the transmission route of HPV? 2. True or false: HPV infection can cause condyloma acuminata, oropharyngeal cancer, cervical cancer, and anal cancer.	To provide a general overview of the topic, by attracting the subjects’ attention with interesting animations.
2	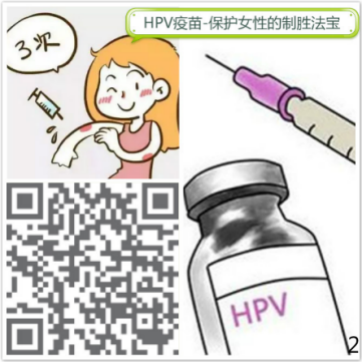	Frequently asked questions and answers about HPV vaccine. For example, how does the HPV vaccine work? What are the recommended ages and populations for vaccination?	1. True or false: (1) HPV vaccine is a cervical cancer vaccine. (2) The best time for HPV vaccination is before sex debut. (3) Women who have been vaccinated against HPV do not need cervical screening.	To convey relevant messages to improve the subjects’ understanding of HPV vaccines and vaccination.
3	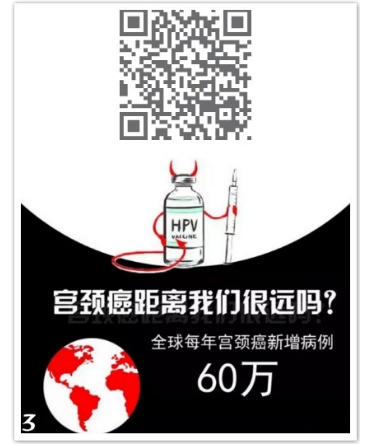	A story of a female movie star who died of cervical cancer. Then, a case of a woman who missed HPV vaccination and cervical cancer screening, reached the terminal stage of cancer right after being found symptomatic, and died after painful treatment. Finally, the relevant facts about the screening, treatment, and prognosis of cervical cancer.	1. True or false: Persistent HPV infection may cause cervical cancer. 2. What is the recommended age for cervical cancer screening?	To use real-life experiences to stimulate the participants’ fear and susceptibility to cervical cancer and arouse their desire to be vaccinated against HPV.
4	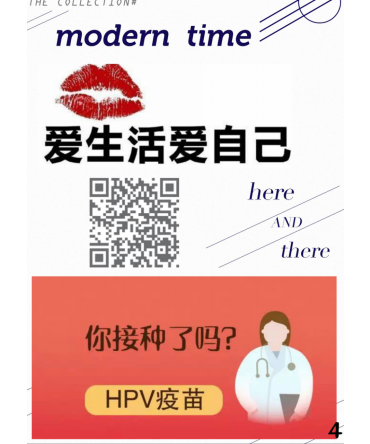	Risk factors and early symptoms of cervical cancer, and ways to prevent and control cervical cancer.	1. Regarding the high-risk factors of cervical cancer, which of the following is wrong? A. Premature birth and fertility B. Premature sex C. Hormone replacement therapy D. Sexual disorder	To call for HPV vaccination and regular cervical cancer screening, in order to arouse the desire of participants to get vaccinated against HPV.
5	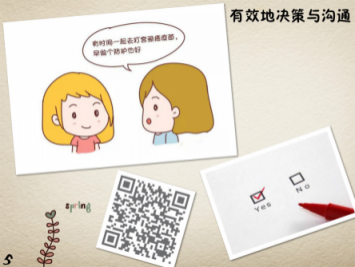	The concept and practice of decision-making and effective communication.	1. Application information: Please select the views that support HPV vaccination (multiple choices) A. I am at risk of contracting HPV. B. I am afraid of needle tingling. C. HPV infection will affect my daily life. D. The price of vaccines is too expensive.	To make firm the participants’ determination to receive HPV vaccines, and communicate effectively with parents and friends to obtain support.
6	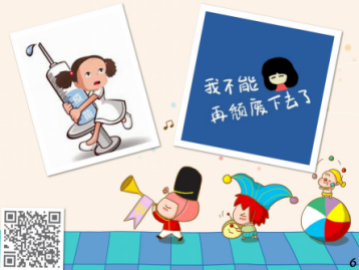	The concept and function of self-efficacy, evaluation of self-efficacy, and ways to improve self-efficacy.	What are the ways to improve self-efficacy (multiple choices)? 1. Try to stick to a good habit. 2. Concentrate on what you do. 3. Evaluate the pros and cons and make a decision. 4. Self-discipline and self-motivation.	To provide guidance on how to improve self-efficacy and turn the idea of HPV vaccination into action.
7	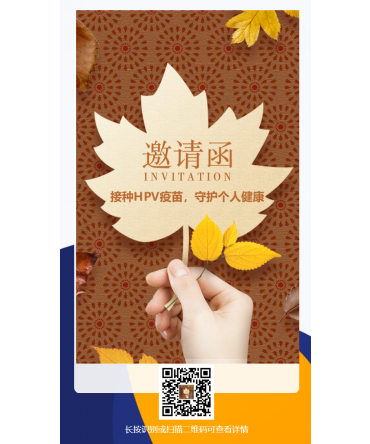	Provide participants with the objective skills needed for HPV vaccination in the form of invitation letters designed with HTML5 front-end technology, such as appointment platform, price, vaccination venue, etc.	The last page is linked to the online questionnaire after the intervention.	To provide detailed objective information required for HPV vaccination, making HPV vaccination more accessible and convenient.

^a^HPV: human papillomavirus.

### Waitlist Control Group

The study was carried out when COVID-19 broke out worldwide at the beginning of 2020. In order to increase the compliance of the control group and reduce the probability of compromising the blind nature of the study, the control group was given 7 days of information on COVID-19 prevention, which was organized and presented in the same format and platform as the educational materials in the intervention group. At the end of the study, the waitlist control group received the same educational materials as the intervention group.

### Outcomes and Measurements

The internet-based questionnaire surveys were administered at 4 time points (baseline, immediately after the intervention, and 1 month and 3 months after the intervention), and participants were given notebooks and pens with the logo of the research institution as incentives.

Background characteristics, including age, major in college, ethnicity, residence, parental residence, education and marital status, monthly living expenses (RMB), family/friends with any cancer, ever received sexual education, currently in a romantic relationship, sexual debut, and attitude toward premarital sex, were measured in this study. These variables are necessary information, basic information, or information related to HPV vaccination as shown in previous literature [30].

The primary outcome measures were self-reported willingness to undergo HPV vaccination and uptake of HPV vaccination. At baseline and each follow-up, the participants were asked “Are you willing to get the HPV vaccine in the future?” and “Have you been vaccinated against HPV?” with “Yes” and “No” response options. It should be noted that there were some differences in the way we asked questions regarding the willingness to undergo HPV vaccination. The 3-month questioning time frame was in the “future,” while the baseline and other 2 follow-up questioning time frames were “within the study period.” The differences in outcomes are detailed in the Results section.

The secondary outcome measures were the information/knowledge, motivation, and behavioral skills regarding HPV vaccination, which were designed based on the IMB model. Among them, the information part consisted of 11 questions. For example, “HPV is related to the development of cervical cancer,” with answer options “Agree,” “Disagree,” and “Do not know” (Cronbach α=.78) [30-33]. Motivation for vaccination was measured by 19 questions. For example, “Getting vaccinated for HPV will help protect me from HPV infection” (Cronbach α=.71) [31,34-36]. Behavioral skills were measured by 10 questions. For example, “I feel confident in my ability to get vaccinated for HPV, even if it is expensive” (Cronbach α=.88) [33,35]. The answers for these items were measured on a 5-point Likert scale (1=strongly disagree, 2=disagree, 3=neither disagree nor agree, 4=agree, and 5=strongly agree). A description of the baseline results for this study has been published [37].

In the 3rd month after the intervention, the participants were asked about their perceptions of the barriers and facilitators of HPV vaccination, such as the reasons for not receiving the HPV vaccine; choices of the HPV vaccine; opinions on promoting HPV vaccination in China; and willingness to receive the HPV vaccine under different scenarios.

### Ethics Approval

This study was approved by the Institutional Review Board of the Chinese Center for Disease Control and Prevention on October 24, 2019 (approval number: 201918-01).

### Statistical Analysis

IBM SPSS Statistics 23.0 (IBM Corp) was used to process the data and conduct statistical comparisons between the intervention and control groups. Independent *t* tests and chi-square tests were used to compare the distributions of the continuous and categorical variables, respectively. Analyses were conducted based on an intention-to-treat approach, and statistical significance was set at *P*<.05 (2-sided). The effects of the IMB model–based intervention on knowledge, motivation, behavioral skills, and willingness regarding HPV vaccination were examined using generalized estimating equations (GEEs). We included significant variables in baseline chi-square analysis, and assessed group (intervention and control), time (baseline, immediately after the intervention, and 1 month and 3 months after the intervention), and time × group interaction, with the time × group interaction indicating a differential change by group from baseline to the end of the trial.

## Results

### Overview

**Figure 1:**
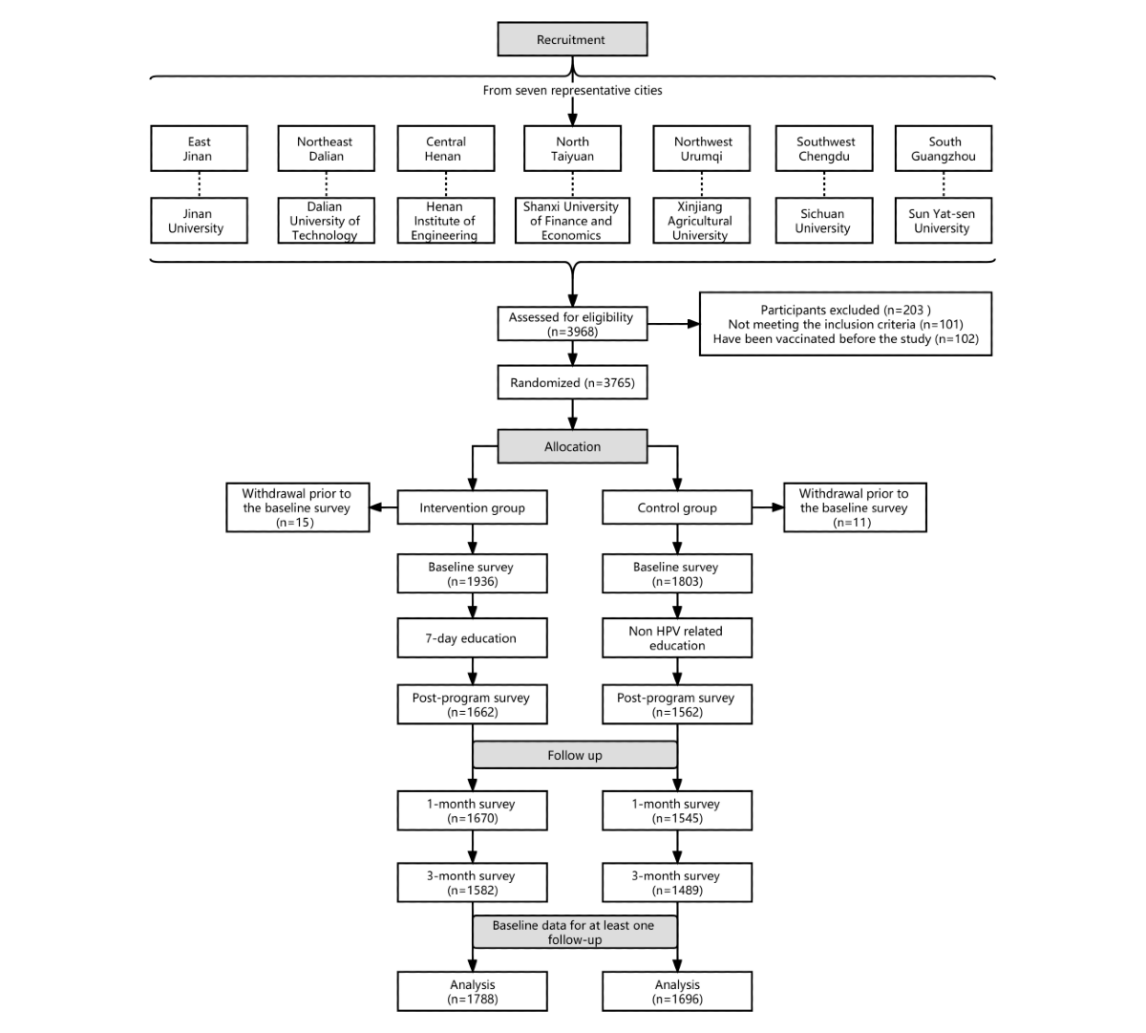
The study flowchart.

The flowchart for participant recruitment is presented in Figure 1. From February 2020 to March 2020, a total of 4051 female college students were recruited and screened, among whom 83 refused to participate in the study, 101 did not meet the inclusion criteria, and 102 reported that they had been vaccinated with the HPV vaccine prior to the study. Among the participants who met the inclusion criteria, the HPV vaccination rate was 2.6% (102/3867). A total of 3765 participants signed the informed consent and completed the baseline questionnaire (T-baseline). However, 26 participants were not grouped and withdrew from the study. There were eventually 1936 participants in the intervention group and 1803 participants in the control group. A total of 3224 (86.2%) participants completed the postintervention questionnaire (T-postintervention). In the intervention group, 1662 participants completed the IMB model–based intervention, and 1562 participants in the control group received non-HPV–related content in the same period. In addition, a total of 3215 (86.0%) participants (1670 in the intervention group and 1545 in the control group) completed the follow-up assessment 1 month after the intervention (T-1 month). Finally, a total of 3071 (82.1%) participants completed the 3-month follow-up evaluation (T-3 months), including 1582 and 1489 participants in the intervention and control groups, respectively. The proportions of participants in the intervention group and control group who completed the T-postintervention assessment were 85.9% (1662/1936) and 86.6% (1562/1803), respectively, and the difference was not statistically significant (*P*=.49). The completion rates of the T-1 month assessment in the intervention and control groups were 86.2% (1670/1936) and 86.6% (1562/1803), respectively, and the difference was not statistically significant (*P*=.62). The completion rates of the T-3 months assessment in the intervention and control groups were 81.7% (1582/1936) and 82.6% (1489/1803), respectively, with no statistically significant difference (*P*=.49).

### Participant Characteristics

The participants with at least one follow-up record were included in our analysis. A total of 3484 respondents were identified, and the mean age was 19.12 (SD 0.73) years. Over half of the respondents (1848/3484, 53.0%) were majoring in Arts in college, and 87.7% (3057/3484) were Han Chinese. Most of the respondents (2207/3484, 63.4%) and their parents (2189/3484, 62.8%) lived in urban areas during the study period. Regarding the educational background of the parents, less than half (1591/3484, 45.7%) of them had a junior high school or lower education, and only 24.6% (856/3484) of the parents had a college degree or above. Most of the respondents’ parents (2819/3484, 80.9%) were married at the time of the research, while 6.9% (239/3484) were divorced, 9.8% (340/3484) were unmarried cohabiting, and 2.5% (86/3484) were widowed. Overall, 71.3% (2483/3484) had monthly living expenses ranging from 1000 to 2000 yuan (US$ 144.35 to 288.71), and about a third (842/3484, 24.2%) of all respondents had relatives or friends with cancer. Moreover, 79.2% (2758/3484) of the respondents self-reported having ever received sexual education, while 16.9% (590/3484) were currently in a romantic relationship. Only 2.8% (97/3484) had sexual debut, while 37.6% (1310/3484) said they could accept premarital sex. As shown in Table 2, both groups were comparable in terms of their sociodemographic characteristics, except previous sexual intercourse, for which there were more participants in the intervention group than in the control group.

**Table 2:** Baseline characteristics of the participants.

Variable		All (N=3484)	Intervention group (N=1788)	Control group (N=1696)	*χ*^2^ (df)	*P* value
Age (years), mean (SD)		19.12 (0.73)	19.12 (0.72)	19.11 (0.73)	0.34^a^ (3482)	.73
**Major in college, n (%)**					0.73 (1)	.39
	Arts	1848 (53.0)	961 (53.8)	887 (52.3)		
	Science	1636 (47.0)	827 (46.3)	809 (47.7)		
**Ethnicity, n (%)**					0.25 (1)	.62
	Han	3057 (87.7)	1564 (87.5)	1493 (88.0)		
	Other	427 (12.3)	224 (12.5)	203 (12.0)		
**Location, n (%)**					1.32 (1)	.25
	Urban	2207 (63.4)	1149 (64.3)	1058 (62.4)		
	Rural	1277 (36.7)	639 (35.7)	638 (37.6)		
**Parental location, n (%)**					1.05 (1)	.31
	Urban	2189 (62.8)	1138 (63.7)	1051 (62.0)		
	Rural	1295 (37.2)	650 (36.4)	645 (38.0)		
**Parental education, n (%)**					0.97 (2)	.62
	Junior high school or less	1591 (45.7)	1428 (79.9)	1391 (82.0)		
	Senior high school or technical secondary school	1037 (29.8)	120 (6.7)	119 (7.0)		
	College or more	856 (24.6)	189 (10.6)	151 (8.9)		
**Marital status of parents, n (%)**					5.29 (3)	.15
	Married	2819 (80.9)	51 (2.9)	35 (2.1)		
	Divorced/separated	239 (6.9)	831 (46.5)	760 (44.8)		
	Unmarried cohabiting	340 (9.8)	524 (29.3)	513 (30.3)		
	Widowed	86 (2.5)	433 (24.2)	423 (24.9)		
**Monthly living expenses (RMB)^b^, n (%)**					5.49 (2)	.06
	<1000	720 (20.7)	392 (21.9)	328 (19.3)		
	1000-2000	2483 (71.3)	1243 (69.5)	1240 (73.1)		
	>2000	281 (8.1)	153 (8.6)	128 (7.6)		
**Family/friends with any cancer, n (%)**					0.78 (1)	.38
	Yes	842 (24.2)	421 (23.6)	421 (24.8)		
	No	2642 (75.8)	1367 (76.5)	1275 (75.2)		
**Ever received sexual education, n (%)**					0.40 (1)	.53
	Yes	2758 (79.2)	1423 (79.6)	1335 (78.7)		
	No	726 (20.8)	365 (20.4)	361 (21.3)		
**Currently in a romantic relationship, n (%)**					0.38 (1)	.54
	Yes	590 (16.9)	296 (16.6)	294 (17.3)		
	No	2894 (83.1)	1492 (83.5)	1402 (82.7)		
**Had sexual debut, n (%)**					4.43 (1)	.04^c^
	Yes	97 (2.8)	60 (3.4)	37 (2.2)		
	No	3387 (97.2)	1728 (96.6)	1659 (97.8)		
**Attitude toward premarital sex, n (%)**					0.29 (1)	.59
	Yes	1310 (37.6)	680 (38.0)	630 (37.2)		
	No	2174 (62.4)	1108 (62.0)	1066 (62.9)		

^a^Analysis for *t* test.

^b^A currency exchange rate of 1 RMB=US $0.14435 is applicable.

^c^Significant *P*<.05.

### Evaluation of the Intervention

Baseline differences were discovered for previous sexual intercourse alone, and as such, all subsequent analyses were adjusted for previous sexual experience (Table 3).

**Table 3:** Generalized estimating equation model of willingness and the 3 dimensions of the information-motivation-behavioral skills model regarding human papillomavirus vaccination between the intervention and control groups.

Variable^a^		Willingness to receive the HPV^b^ vaccine			Information			Motivation			Behavioral skills	
		*β*	*P* value	*β*		*P* value	*β*		*P* value	*β*		*P* value
**Sexual debut**												
	No	Reference		Reference			Reference			Reference		
	Yes	1.025	<.001^c^	1.037		<.001^c^	0.171		<.001^c^	0.137		.01^c^
**Time^d^**												
	T-baseline	Reference		Reference			Reference			Reference		
	T-postintervention	−0.019	.67	0.391		<.001^c^	−0.055		<.001^c^	−0.029		.006^c^
	T-1 month	−0.055	.27	0.628		<.001^c^	−0.065		<.001^c^	−0.043		<.001^c^
	T-3 months	1.476	<.001^c^	0.762		<.001^c^	−0.035		<.001^c^	−0.017		.23
**Group**												
	Control	Reference		Reference			Reference			Reference		
	Intervention	0.042	.56	0.103		.26	0.002		.84	0.011		.52
**Time^d^ × group **												
	T-baseline × intervention/control	Reference		Reference			Reference			Reference		
	T-postintervention × intervention/control	0.320	<.001^c^	2.202		<.001^c^	0.115		<.001^c^	0.104		<.001^c^
	T-1 month × intervention/control	0.183	.01^c^	1.892		<.001^c^	0.085		<.001^c^	0.062		<.001^c^
	T-3 months × intervention/control	0.140	.13	1.688		<.001^c^	0.053		<.001^c^	0.059		.003^c^

^a^The generalized estimating equation model was adjusted with the significant variable in the chi-square analysis of baseline data, namely “sexual debut.”

^b^HPV: human papillomavirus.

^c^Significant *P*<.05.

^d^Time points: baseline (T-baseline), immediately after the intervention (T-postintervention), 1 month after the intervention (T-1 month), and 3 months after the intervention (T-3 months).

#### Willingness to Receive the HPV Vaccine

The GEE revealed the simple effects of time (T-3 months vs T-baseline, *β*=1.476; *P*<.001) and a significant time × group interaction for T-postintervention vs T-baseline (*β*=0.320; *P*<.001) and T-1 month vs T-baseline (*β*=0.183; *P*=.011), but no group effect (intervention group vs control group; *β*=0.042, *P*=.56). Compared with the control group, the intervention group showed a significant increase in the willingness to undergo HPV vaccination immediately and 1 month after the intervention.

#### HPV Information

The GEE revealed the simple effects of time (T-postintervention, T-1 month, and T-3 months vs T-baseline; *β*=0.391, 0.628, and 0.762, respectively; *P*<.001) and a significant time × group interaction for T-postintervention, T-1 month, and T-3 months vs T-baseline (*β*=2.202, 1.892, and 1.688, respectively; *P*<.001), but no group effect (intervention group vs control group; *β*=0.103; *P*=.26). Compared with the control group, the intervention group showed a significant increase in HPV information after the intervention.

#### Motivation for HPV Vaccination

The GEE revealed the simple effects of time (T-postintervention, T-1 month, and T-3 months vs T-baseline; *β*=−0.055, −0.065, and −0.035, respectively; *P*<.001) and a significant time × group interaction for T-postintervention, T-1 month, and T-3 months vs T-baseline (*β*=0.115, 0.085, and 0.053, respectively; *P*<.001), but no group effect (intervention group vs control group; *β*=0.002; *P*=.84). Compared with the control group, the intervention group showed a significant increase in motivation for HPV vaccination after the intervention.

#### Behavioral Skills for HPV Vaccination

The GEE revealed the simple effects of time for T-postintervention vs T-baseline (*β*=−0.029; *P*=.006) and for T-1 month vs T-baseline (*β*=−0.043; *P*<.001). A significant time × group interaction was found for T-postintervention and T-1 month vs T-baseline (*β*=0.104 and 0.062, respectively; *P*<.001) and for T-3 months vs T-baseline (*β*=0.059; *P*=.003), but there was no group effect (intervention group vs control group; *β*=0.011; *P*=.52). Compared with the control group, the intervention group showed a significant increase in behavioral skills for HPV vaccination after the intervention.

### Intervention Effects on the Willingness to Undergo HPV Vaccination and Practice of HPV Vaccination

Table 4 shows the main effect of the intervention on the willingness to undergo HPV vaccination from baseline to follow-up. At baseline, the willingness rates to undergo HPV vaccination in the intervention and control groups were 33.33% and 31.96%, respectively, and there was no significant difference between the 2 groups (*χ*^2^_1_=0.75; *P*=.39). Immediately after the intervention, the willingness rates to undergo HPV vaccination in the intervention and control groups were 40.39% and 31.56%, respectively, with statistically significant differences between the 2 groups and within the intervention group from T-baseline to T-postintervention (*χ*^2^_1_=27.11; *P*<.001; and *χ*^2^_1_=15.43; *P*<.001, respectively). At T-1 month, the willingness rates to receive the HPV vaccine in the intervention and control groups were 35.65% and 31.34%, respectively. At this point, the difference between the 2 groups was statistically significant (*χ*^2^_1_=6.64; *P*=.01), but there was no significant difference within the intervention group from T-baseline to T-1 month (*χ*^2^_1_=1.40; *P*=.24). At T-3 months, the willingness rates to undergo HPV vaccination in the intervention and control groups were 70.59% and 66.17%, respectively, with statistically significant differences between the 2 groups and within the intervention group from T-baseline to T-3 months (*χ*^2^_1_=6.81; *P*=.01; and *χ*^2^_1_=368.59; *P*<.001, respectively).

At different time points, the HPV vaccination rates in the intervention and control groups were as follows: T-postintervention, 0.48% and 0.19%, respectively (*χ*^2^_1_=1.98; *P*=.16); T-1 month, 0.72% and 0.65%, respectively (*χ*^2^_1_=0.06; *P*=.81); T-3 months, 2.21% and 1.75%, respectively (*χ*^2^_1_=0.86; *P*=.36).

**Table 4:** Main effect of the intervention on the willingness to undergo human papillomavirus vaccination from baseline to follow-up.

Variable and time^a^			Intervention group				*χ*^2^^b^ (df)		*P* value^b^		Control group					*χ*^2^^c^ (df)			*P* value^c^
			Yes, n/N (%)		No, n/N (%)						Yes, n/N (%)		No, n/N (%)						
**Willingness to receive the HPV^d^ vaccine**																			
	T-baseline^e^	596/1788 (33.3)		1192/1788 (66.7)		N/A^f^		N/A		542/1696 (32.0)		1154/1696 (68.0)		0.75 (1)			.39		
	T-postintervention^e^	668/1654 (40.4)		986/1654 (59.6)		15.43 (1)		<.001^g^		492/1559 (31.6)		1067/1559 (68.4)		27.11 (1)			<.001^g^		
	T-1 month^e^	591/1658 (35.7)		1067/1658 (64.3)		1.40 (1)		.24		481/1535 (31.3)		1054/1535 (68.7)		6.64 (1)			.01^g^		
	T-3 months^h^	1092/1547 (70.6)		455/1547 (29.4)		368.59 (1)		<.001^g^		968/1463 (66.2)		495/1463 (33.8)		6.81 (1)			.01^g^		

^a^Time points: baseline (T-baseline), immediately after the intervention (T-postintervention), 1 month after the intervention (T-1 month), and 3 months after the intervention (T-3 months).

^b^Comparison between baseline and different follow-up time points in the intervention group.

^c^Comparison between the intervention and control groups at baseline and each follow-up time point.

^d^HPV: human papillomavirus.

^e^Are you willing to get the HPV vaccine in recent months?

^f^N/A: not applicable.

^g^Significant *P*<.05.

^h^Are you willing to get the HPV vaccine in the future?

### Intervention Effects on IMB Model Variables

#### HPV-Related Information

**Figure 2:**
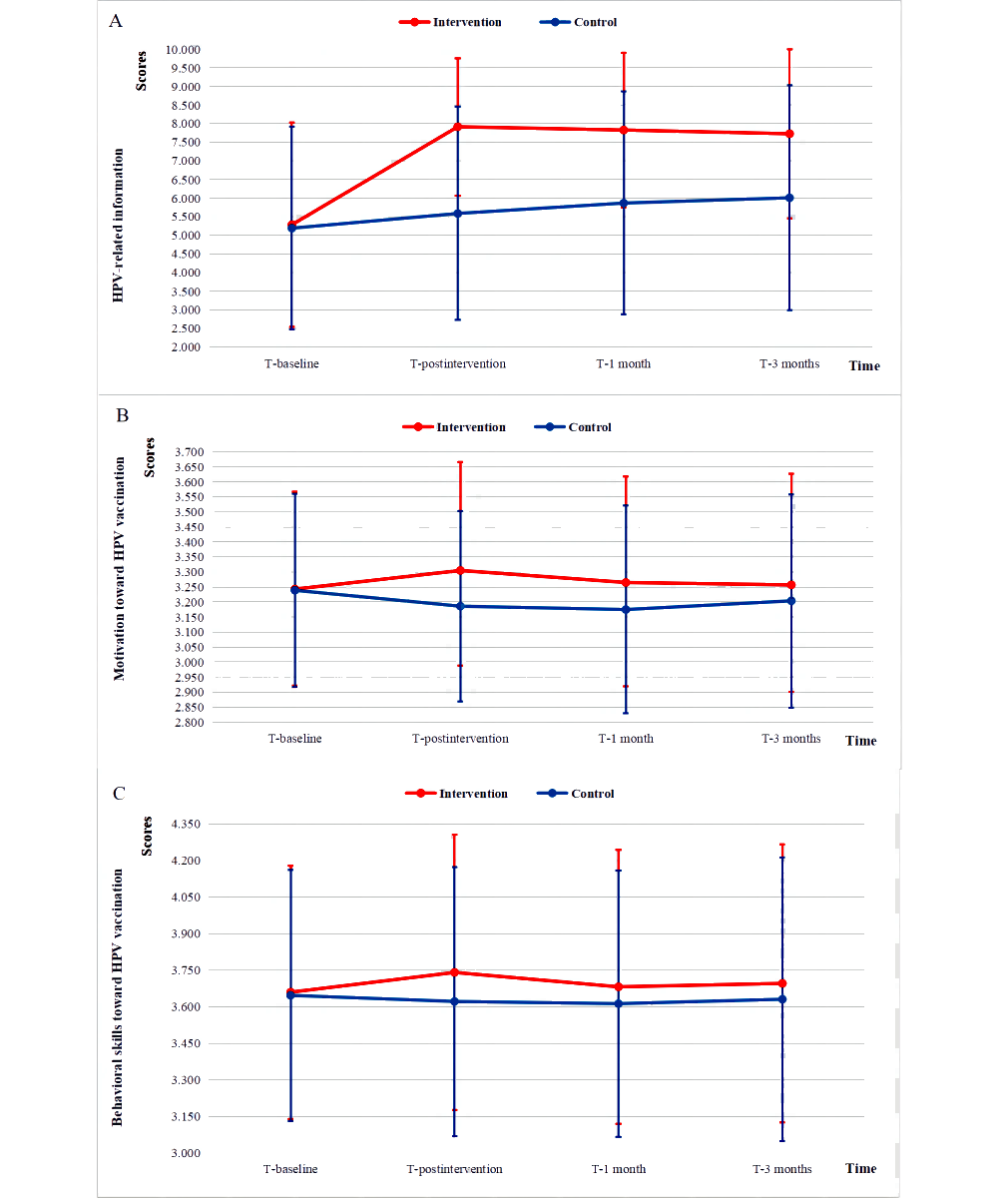
Trends of the mean scores of information (A), motivation (B), and behavioral skills (C) regarding human papillomavirus (HPV) vaccination in the intervention and control groups over time.

Compared with the control group, the participants in the intervention group demonstrated a significant improvement in knowledge scores from baseline to any posttest time point (*P*<.001). In addition, there were significant changes in knowledge scores from baseline to any posttest time point (*P*<.001) within the intervention group (Figure 2A; Table 5).

**Table 5:** Main effect of the intervention on the mean scores of the information-motivation-behavioral skills model constructs regarding human papillomavirus vaccination from baseline to follow-up.

IMB^a^ model constructs and time^b^			Intervention group, mean score (SD)		Mean difference within the group (95% CI)		*t*^c^ (df)		*P* value^c^		Control group, mean score (SD)		Mean difference between groups (95% CI)		*t*^d^ (df)		*P* value^d^
**Information**																	
	T-baseline	5.29 (2.74)		N/A^e^		N/A		N/A		5.20 (2.72)		0.93 (−0.09 to 0.27)		1.00 (3482)		.32	
	T-postintervention	7.92 (1.84)		2.58 (2.43 to 2.74)		32.70 (3448)		<.001^f^		5.59 (2.86)		2.33 (2.16 to 2.49)		27.29 (3222)		<.001^f^	
	T-1 month	7.83 (2.07)		2.51 (2.34 to 2.67)		30.52 (3456)		<.001^f^		5.87 (2.99)		1.96 (1.78 to 2.14)		21.46 (3213)		<.001^f^	
	T-3 months	7.73 (2.27)		2.40 (2.22 to 2.57)		27.25 (3368)		<.001^f^		6.01 (3.02)		1.72 (1.53 to 1.91)		17.76 (3069)		<.001^f^	
**Motivation**																	
	T-baseline	3.24 (0.32)		N/A		N/A		N/A		3.24 (0.32)		0.00 (−0.02 to 0.03)		0.39 (3482)		.70	
	T-postintervention	3.31 (0.36)		0.06 (0.03 to 0.08)		4.81 (3448)		<.001^f^		3.19 (0.32)		0.12 (0.19 to 0.14)		10.00 (3222)		<.001^f^	
	T-1 month	3.27 (0.35)		0.02 (−0.01 to 0.04)		1.50 (3456)		.13		3.18 (0.35)		0.09 (0.07 to 0.11)		7.31 (3213)		<.001^f^	
	T-3 months	3.26 (0.37)		0.01 (−0.02 to 0.03)		0.59 (3368)		.56		3.20 (0.36)		0.05 (0.03 to 0.08)		4.01 (3069)		<.001^f^	
**Behavioral skills**																	
	T-baseline	3.66 (0.52)		N/A		N/A		N/A		3.65 (0.52)		0.01 (−0.02 to 0.05)		0.74 (3482)		.46	
	T-postintervention	3.74 (0.57)		0.07 (0.03 to 0.10)		3.70 (3448)		<.001^f^		3.62 (0.55)		0.12 (0.08 to 0.16)		6.04 (3222)		<.001^f^	
	T-1 month	3.68 (0.56)		0.01 (−0.03 to 0.05)		0.60 (3456)		.55		3.61 (0.55)		0.067 (0.03 to 0.11)		3.54 (3213)		<.001^f^	
	T-3 months	3.70 (0.57)		0.02 (−0.02 to 0.05)		0.85 (3368)		.39		3.63 (0.58)		0.07 (0.02 to 0.11)		3.16 (3069)		<.001^f^	

^a^IMB: information-motivation-behavioral skills.

^b^Time points: baseline (T-baseline), immediately after the intervention (T-postintervention), 1 month after the intervention (T-1 month), and 3 months after the intervention (T-3 months).

^c^Comparison between baseline and different follow-up time points in the intervention group.

^d^Comparison between the intervention and control groups at baseline and each follow-up time point.

^e^N/A: not applicable.

^f^Significant *P*<.05.

#### Motivation for HPV Vaccination

Compared with the control group, the participants in the intervention group demonstrated a significant improvement in motivation from baseline to any posttest time point (*P*<.001). However, in the intervention group, there were no significant changes in motivation scores from T-baseline to T-1 month or T-3 months (*P*=.13 and *P*=.56, respectively), except for a significant change from T-baseline to T-postintervention (*P*<.001) (Figure 2B; Table 5).

#### Behavioral Skills for HPV Vaccination

Similar to the trend for motivation, compared with the control group, the participants in the intervention group demonstrated a significant improvement in behavioral skills from baseline to any posttest time point (*P*<.001). However, in the intervention group, there were no significant changes in scores of behavioral skills from T-baseline to T-1 month or T-3 months (*P*=.55 and *P*=.39, respectively), except for a significant change from T-baseline to T-postintervention (*P*<.001) (Figure 2C; Table 5).

### Perceptions of the Barriers and Facilitators of Receiving the HPV Vaccine

Table 6 shows that 2060 (68.4%) participants were willing to undergo HPV vaccination and 2788 (90.52%) participants would recommend HPV vaccination to their relatives and friends. The reasons for not undergoing or recommending HPV vaccination included “worried about its side effects” (471/1694, 27.8%), “expensive” (433/1694, 25.6%), “unsure of its safety and effectiveness” (406/1694, 24.0%), “lack of knowledge about the HPV vaccine” (318/1694, 18.8%), “have no sexual behavior” (279/1694, 16.5%), and “possible needle injury” (178/1694, 10.5%).

**Table 6:** Perceptions of the barriers and facilitators of receiving the human papillomavirus vaccine.

Variable				All, n (%)		Intervention group, n (%)		Control group, n (%)
**Reasons for not receiving the HPV^a^ vaccine**								
	**Willing to undergo HPV vaccination (N=3010; I^b^: 1547, C^c^: 1463)**							
		Yes	2060 (68.4)		1092 (70.6)		968 (66.2)	
		No	950 (31.6)		455 (29.4)		495 (33.8)	
	**Willing to recommend HPV vaccination (N=3071; I: 1582, C: 1489)**							
		Yes	2780 (90.5)		1447 (91.5)		1333 (89.5)	
		No	291 (9.5)		135 (8.5)		156 (10.5)	
	**Reasons for not undergoing or recommending HPV vaccination (N=1694; I: 863, C: 831)**							
		Do not know much about the HPV vaccine	318 (18.8)		119 (13.8)		199 (24.0)	
		Unsure of its safety and effectiveness	406 (24.0)		171 (19.8)		235 (28.3)	
		Worried about its side effects	471 (27.8)		210 (24.3)		261 (31.4)	
		Possible needle injury	178 (10.5)		82 (9.5)		96 (11.6)	
		Expensive	433 (25.6)		228 (26.4)		205 (24.7)	
		Too many needles	123 (7.3)		68 (7.9)		55 (6.6)	
		No sexual behavior	279 (16.5)		142 (16.5)		137 (16.5)	
		Others	19 (1.1)		14 (1.6)		5 (0.6)	
**Opinions on promoting HPV vaccination (N=3071; I: 1582, C: 1489)**								
	**The promotion of the HPV vaccine will be well accepted**							
		Yes	2760 (89.9)		1425 (90.1)		1335 (89.7)	
		No	311 (10.1)		157 (9.9)		154 (10.3)	
	**The biggest concern about the HPV vaccine**							
		Efficacy/effectiveness	507 (16.5)		259 (16.4)		248 (16.7)	
		Safety	856 (27.9)		436 (27.6)		420 (28.2)	
		Side effects	543 (17.7)		254 (16.1)		289 (19.4)	
		Price	786 (25.6)		425 (26.9)		361 (24.2)	
		Procedure for appointment and HPV vaccination	250 (8.1)		135 (8.5)		115 (7.7)	
		Nothing to worry about	94 (3.1)		57 (3.6)		37 (2.5)	
	**The barriers for promoting HPV vaccination**							
		Insufficient message from the government	1660 (54.1)		821 (51.9)		839 (56.4)	
		Too expensive	2330 (75.9)		1230 (77.8)		1100 (73.9)	
		Not covered by medical insurance	1718 (55.9)		874 (55.3)		844 (56.7)	
		Not included in national immunization programs	1587 (51.7)		806 (51.0)		781 (52.5)	
		Inconvenient to schedule vaccination	1218 (39.7)		611 (38.6)		607 (40.8)	
		Insufficient experience of doctors	770 (25.1)		360 (22.8)		410 (27.5)	
		Vaccine hesitancy	769 (25.0)		374 (23.6)		395 (26.5)	
		HPV vaccine–related stigma	550 (17.9)		266 (16.8)		284 (19.1)	
	**How to promote HPV vaccination**							
		Improve doctor’s recommendation	2341 (76.2)		1189 (75.2)		1152 (77.4)	
		Media publicity	1793 (58.4)		908 (57.4)		885 (59.4)	
		Make it convenient to schedule vaccination	1994 (64.9)		1036 (65.5)		958 (64.3)	
		Provide the vaccine at a reasonable price	2453 (79.9)		1271 (80.3)		1182 (79.4)	
**The most preferable type of HPV vaccine (N=3010; I: 1547, C: 1463)**								
	2-valent HPV (Cervarix, GlaxoSmithKline )		552 (18.3)		287 (18.6)		265 (18.1)	
	4-valent HPV (Gardasil, Merck and Co, Inc)		425 (14.1)		224 (14.5)		201 (13.7)	
	9-valent HPV (Gardasil 9, Merck and Co, Inc)		947 (31.5)		489 (31.6)		458 (31.3)	
	2-valent HPV (Cecolin, Innovax)		1087 (36.1)		548 (35.4)		539 (36.8)	
**Willingness to receive the HPV vaccine in the future under different scenarios (N=3010; I: 1547, C: 1463)**								
	**If the price is affordable**							
		Yes	2839 (94.3)		1465 (94.7)		1374 (93.9)	
		No	171 (5.7)		82 (5.3)		89 (6.1)	
	**If it is covered by medical insurance**							
		Yes	2862 (95.1)		1471 (95.1)		1391 (95.1)	
		No	148 (4.9)		76 (4.9)		72 (4.9)	
	**If it is included in national immunization programs**							
		Yes	2887 (95.9)		1492 (96.4)		1395 (95.4)	
		No	123 (4.1)		55 (3.6)		68 (4.6)	

^a^HPV: human papillomavirus.

^b^I: number of participants in the intervention group.

^c^C: number of participants in the control group.

Among the 3071 complaint participants, most of them (2760/3071, 89.9%) believed that the promotion of the HPV vaccine would be well accepted. The major concerns about the HPV vaccine were “safety” (856/3071, 27.9%) and “price” (786/3071, 25.6%), followed by “side effects” (543/3071, 17.7%) and “efficacy” (507/3071, 16.5%). In addition, there were 250 (8.1%) participants concerned about the “procedure for appointment and uptake of the HPV vaccine,” and only 94 (3.1%) participants did not express any concerns. Regarding the barriers for promoting HPV vaccination, “too expensive” (2330/3071, 75.9%), “not being covered by medical insurance” (1718/3071, 55.9%), “insufficient message from the government” (1660/3071, 54.1%), and “not being included in national immunization programs” (1587/3071, 51.7%) were major concerns. Each of the measures to promote HPV vaccination in China received more than 50% agreement from the participants, including “reasonable price” (2453/3071, 79.9%), “doctors’ recommendation” (2341/3071, 76.2%), “be convenient to schedule vaccination” (1994/3071, 64.9%), and “media publicity” (1793/3071, 58.4%).

Interestingly, the most preferable type of HPV vaccine we found was the domestically produced 2-valent HPV vaccine (1087/3010, 36.1%), followed by the 9-valent HPV vaccine (947/3010, 31.5%), the 2-valent HPV vaccine (552/3010, 18.3%), and the 4-valent HPV vaccine (425/3010, 14.1%), which were all produced abroad.

The willingness rate to receive the HPV vaccine was 94.3% (2839/3010) if the price was affordable. In addition, it increased to 95.1% (2862/3010) if the HPV vaccination was covered by medical insurance, and it increased to 95.9% (2887/3010) if the HPV vaccination was included in national immunization programs, compared with 68.4% (2060/3010) unconditional willingness.

## Discussion

### Principal Findings

#### Overview

This study investigated the effectiveness of an IMB model–based online education program for HPV vaccination among female students from 7 universities in mainland China. To our knowledge, this is the first study in China to evaluate the effect of an online health education program guided by a health behavioral theory on improving the awareness, attitudes, and willingness regarding HPV vaccination with a randomized intervention and multiple follow-ups.

#### Changes in the Willingness to Undergo HPV Vaccination

After the education program, there was a significant increase in the willingness to be vaccinated against HPV from baseline in the intervention group. Moreover, the willingness to undergo HPV vaccination was significantly higher in the intervention group than in the control group at the end of the study. In line with the findings of previous studies [21,38,39], the effect of the health education program peaked right after the intervention and then gradually diminished over time, but finally, the residual effect of the health education program could still be observed. In this study, participants’ motivation toward HPV vaccination was obviously activated right after the tailored IMB model–based education program, which was consistent with the finding of a previous similar study among impulsive youth [40]. As the passion fades, the participants might begin to think rationally and objectively based on what they had learned about HPV-related issues. They might search for information to expand their knowledge of the HPV vaccine and then try to make an appointment for vaccination. During this process, they are likely to be concerned with the side effects and relatively high price of the HPV vaccine, and might find it difficult to make an appointment for HPV vaccination, which would curb their willingness to receive the vaccine. This study was conducted during the early stage of the COVID-19 pandemic. Due to the constraints of home isolation and traffic control, and the fact that medical resources were tilted toward the prevention and treatment of COVID-19 infection, it was more difficult for the participants to make an appointment and receive the HPV vaccine than usual [41,42]. Therefore, the willingness to be vaccinated against HPV decreased to the baseline level 1 month after the education program. Finally, at the end of the study, participants in the intervention and control groups self-reported a higher level of willingness to be vaccinated against HPV than that at baseline, and the willingness was significantly higher in the intervention group than in the control group. This reminds us that even if education cannot achieve the expected effect in the short term, the seeds of health education will take root and sprout in the participants’ brains, which will trigger thinking in some people and may guide their decision-making in the future. Additionally, these results imply that re-education is necessary to sustain the educational effect.

#### Intervention Effects on IMB Variables

The daily education and matching quizzes in the intervention period, which contained questions in the information part of the questionnaire, probably resulted in a deeper understanding of HPV-related issues in the intervention group compared with the control group, even at 3 months after the education program. In addition, the results also indicated that this study was readily acceptable to the participants in terms of both educational contents and forms, and this coincided with the feedback comments and suggestions from the participants at the end of the study (data are not presented in this manuscript).

Different from the trend of knowledge over time, motivation and behavioral skills regarding HPV vaccination only showed significant differences in the intervention group immediately after the education program. In this study, a 2-day education program addressing the role of preventing HPV infection and negative treatment experiences was given to the participants in the intervention group, which resulted in a large increase in motivation to receive the HPV vaccine among the participants. Similarly, a 3-day education program on decision-making, self-efficacy, and objective skills regarding HPV vaccination was given to the intervention group, which not only guided on the ways to receive financial and emotional support from family and friends, but also clarified the appointment process of HPV vaccination and other related issues of concern. The tailored factual information of this study covered almost all of the possible difficulties that the students might encounter after they decided to receive the HPV vaccine. Previous studies [43-45] showed that compared with awareness and attitudes toward health behaviors, the adoption of health behaviors usually had a certain time lag and needed to be gradually realized in long-term positive practice. In addition, when the scores of the intervention and control groups were compared, the intervention group scored better than the control group in each survey after the education program. The results of this study suggest that although the 7-day education program might not have been sufficient to significantly improve motivation and behavioral skills regarding HPV vaccination in the intervention group at the end of the study, it can maintain participants’ motivation and behavioral skills at certain levels and prevent them from falling.

#### Perceptions of the Barriers and Facilitators of Receiving the HPV Vaccine

About two-thirds of the participants in our study were willing to be vaccinated against HPV, which is higher than the proportion reported in previous studies [46-48], and 90.5% of them were likely to recommend the HPV vaccine to their friends or relatives, which suggests that most of them agreed with the benefits of the HPV vaccine, but had more concerns when it came to themselves, such as concerns about the safety, effectiveness, and possible side effects of the HPV vaccine [49]. In addition, about a quarter of the participants in the control group believed that a lack of knowledge about HPV-related issues was holding them back from receiving the HPV vaccine, which suggests that importance should be given to improving the knowledge and awareness of HPV vaccination among target populations. Additionally, the government should take efforts to make the HPV vaccine affordable and accessible for most college students; to ensure the supply of the domestically produced HPV vaccine, which is popular among female college students; and to promote the coverage of HPV vaccination by medical insurance or its inclusion in national immunization programs as soon as possible. Furthermore, the results of this study suggest that in order to promote HPV vaccination, media publicity of the HPV vaccine should be strengthened nationwide, HPV-related information should be added to the in-service education of doctors, and doctors should be encouraged to recommend the HPV vaccine to female individuals during the consultation process.

### Strengths and Limitations

This is the first randomized trial to study the effectiveness of an IMB model–guided education program to improve HPV vaccination among female college students in mainland China. The strengths of this study include its multicenter design, the inclusion of a representative and sufficient sample of the target population, and the application of a health behavioral theory. Owing to online education and participant management, our study was carried out as scheduled even during the period of the COVID-19 outbreak. This provided a unique opportunity to make clear statements about the feasibility and convenience of the study. In addition, most of the possible contaminations were avoided owing to home isolation of the participants during the study period. The quality control measures of the study can be considered strengths. They included a professional research design and pilot trial conducted by researchers with public health and epidemiological backgrounds, daily quizzes and regular reminders to improve the maintenance rate during follow-up, and logical questions to ensure the quality of the available data.

Several study limitations must be considered. First, the effectiveness of the study might have been compromised by cluster randomization and a mismatch in the number of participants in the intervention and control groups. In addition, although the IMB model–based intervention showed relatively desirable results, the adverse impacts of the COVID-19 pandemic could not be ignored, which included too much concern about getting COVID-19 and the possible trauma, as well as difficulties in making an appointment and getting vaccinated against HPV. Moreover, the participants were asked to self-report if they had received the HPV vaccine before and within the study; however, the accuracy of these self-reported data could not be verified through the community medical care system. Future studies with randomization at the individual level, a longer follow-up period, and regular repetition of education will allow better exploration of the effects of the intervention.

### Conclusions

Online education based on the IMB model can improve HPV-related knowledge, as well as the motivation, behavioral skills, and willingness regarding HPV vaccination among female college students in the short term; however, its long-term effects may need to be consolidated and maintained by repeated education programs in the future.

## Appendix

Multimedia Appendix 1 CONSORT-eHEALTH checklist.

## Abbreviations

GEE: generalized estimating equation
HPV: human papillomavirus
IMB: information-motivation-behavioral skills
